# Controlling Atomic-Scale Restructuring and Cleaning of Gold Nanogap Multilayers for Surface-Enhanced Raman Scattering Sensing

**DOI:** 10.1021/acssensors.3c00967

**Published:** 2023-07-06

**Authors:** David-Benjamin Grys, Marika Niihori, Rakesh Arul, Sarah May Sibug-Torres, Elle W. Wyatt, Bart de Nijs, Jeremy J. Baumberg

**Affiliations:** NanoPhotonics Centre, Cavendish Laboratory, Department of Physics, University of Cambridge, JJ Thompson Avenue, Cambridge CB3 0HE, U.K.

**Keywords:** cleaning, nanogap, sensing, AuNP aggregates, SERS

## Abstract

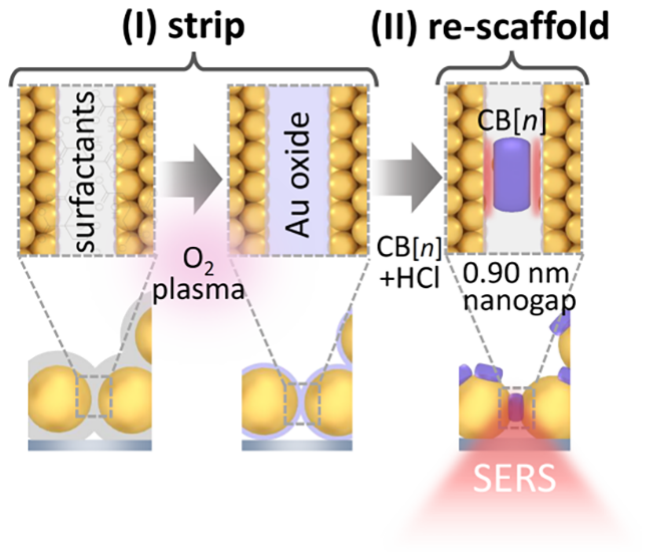

We demonstrate the reliable creation of multiple layers of Au nanoparticles in random close-packed arrays with sub-nm gaps as a sensitive surface-enhanced Raman scattering substrate. Using oxygen plasma etching, all the original molecules creating the nanogaps can be removed and replaced with scaffolding ligands that deliver extremely consistent gap sizes below 1 nm. This allows precision tailoring of the chemical environment of the nanogaps which is crucial for practical Raman sensing applications. Because the resulting aggregate layers are easily accessible from opposite sides by fluids and by light, high-performance fluidic sensing cells are enabled. The ability to cyclically clean off analytes and reuse these films is shown, exemplified by sensing of toluene, volatile organic compounds, and paracetamol, among others.

Surface-enhanced Raman scattering (SERS) is a promising optical sensing technique enabling trace and even single-molecule detection. This is made possible by the electromagnetic field amplification occurring in nanogaps (hotspots) between coinage metal nanostructures. The result is billion-fold enhancement of inelastic Raman scattering from analytes if the metal-insulator-metal spacings are small enough. However, fabricating such thin-film SERS substrates with small gaps, as suited for sensing applications, requires these to be stable, reproducible, easy to fabricate, and have reliable SERS enhancements.^1^ To achieve this, it is imperative that the hotspots are precisely defined because these strongly affect the field enhancements and thus the SERS signal strengths. In addition, surface chemistry, nanoscale geometry, and size need to be tightly controlled as these also affect reproducibility. Top-down approaches such as electron-beam lithography,^2−4^ deep-UV lithography,^5^ focused-ion beam milling,^6,7^ and nanoimprint lithography^8−10^ have been used to fabricate reproducible and scalable SERS substrates with pristine metal surfaces. However, these lithography-based strategies are time-consuming, require high-cost infrastructure, and can only reliably reach gap dimensions >5 nm.^11,12^

Alternatively, bottom-up approaches based on nanoparticle self-assembly offer low-cost, facile fabrication of thin-film SERS substrates. Using template-assisted,^13−15^ evaporative,^16−20^ and interfacial^21−27^ self-assembly, close-packed nanoparticle constructs can be prepared with high spatial uniformity. By selecting the nanoparticle surfactant^17,23,25^ and carefully controlling the self-assembly process,^16,18,19^ inter-nanoparticle gap spacings can be tuned, even down to the sub-nanometer level.^28^ Control of surface chemistry can be problematic for nanoparticle-based substrates, as synthesized nanoparticles often incorporate additional chemicals and surfactants to increase their shelf-life or functionality (either commercially prepared or produced in-house). Surface molecules cannot be fully removed, even by ligand-exchange, causing interference with the target analyte binding association constants^29^ and diminishing the sensitivity to trace analytes by blocking the regions of greatest SERS enhancement. Variation of surfactants between batches and aging of gold nanoparticles (AuNPs) involving adatom morphological changes on the metal facets^30^ can lead to poor uniformity and reproducibility of these substrates.

We present here a simple and reproducible method to efficiently construct films with uniform nanogaps that can be used to sequester and detect small molecules with high levels of specificity. These “multilayer aggregates” (MLaggs) consist of dense-packed single (or bi-) layers of near-spherical AuNPs with precision-controlled nanogap separations defined by rigid molecular scaffolds such as cucurbit[*n*]urils.^29,30^ The AuNPs form a close-packed disordered network but have a well-defined fill fraction, allowing for excellent optical properties due to the consistent sub-nanometer (<1 nm) gap spacing control. Having a few monolayers of AuNPs allows the analyte to diffuse uniformly across the nanogaps and gives capability for reproducible backside illumination.

MLaggs can be directly deposited onto various substrates including glass, Si, PDMS, or Au-coated silicon wafers and integrated into flow systems (see Methods). Once MLaggs are fixed in place, oxygen plasma etching, which is known to break down organic compounds, can be used to strip any molecules (citrate, stabilizing agents, coagulants, contaminants) from the MLagg.^31^ We find here that nanogaps are preserved in the physically supported MLaggs during oxygen plasma cleaning or when flushing analytes from the nanogaps using HCl. This now enables continuously reusable flow sensing systems, which would be unviable for solution aggregate SERS. Using MLaggs, we demonstrate liquid, vapor, and flow sensing, highlighting their exceptional suitability for integration with other devices in a variety of applications spanning from environmental to healthcare monitoring.

## Results and Discussion

### Multilayer Aggregate Preparation and Characterization

**Figure 1:**
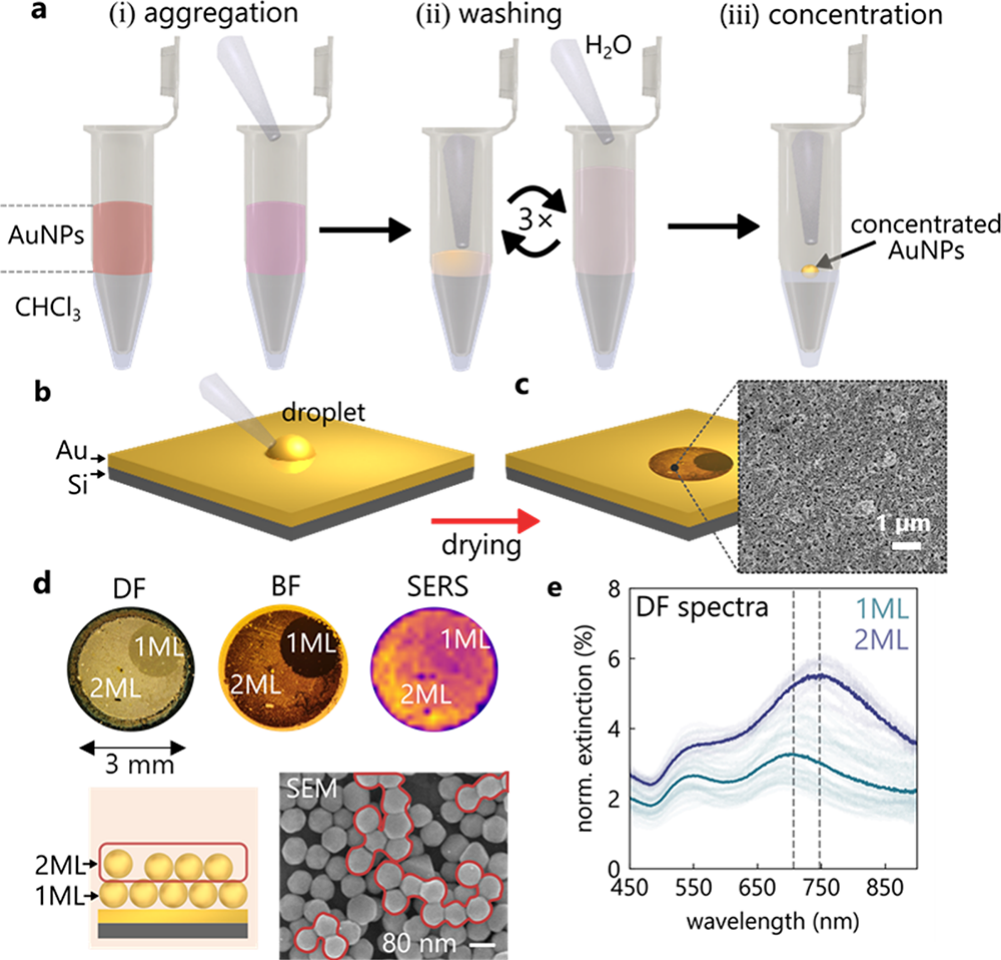
MLagg protocol and characterization. (a) Preparation protocol by (i) partial aggregation of AuNPs in water above CHCl_3_, (ii) salt-removal (“washing”) by repeated replacement of supernatant with DI water, and (iii) final concentration step. (b) Deposition of MLagg droplet on the Au/Si substrate. (c) Dried film and scanning electron microscopy (SEM) showing a dense-packed film of AuNPs. (d) Dark-field (DF), bright-field (BF) images, and SERS map scans showing single (1ML) and double (2ML) layer regions, with close-up SEM confirming the existence of 1ML and 2ML (red outline) layers. (e) DF spectra of 1,2ML regions.

MLagg films are simply prepared in <5 min by partial aggregation of AuNPs (80 nm diameter unless otherwise stated, 15–120 nm also tested) in a two-phase chloroform-water system (Figure 1a). The addition of an aggregating agent (either salts or other ligands) forces AuNPs to the water–air and water–chloroform interfaces (a,i). Removal of the supernatant concentrates AuNPs at the interfaces, visible to the eye as a reflective red-gold film. Three-fold repetition of this washing procedure further increases the AuNP density (ii), leaving a small AuNP droplet (∼10 μL of residual supernatant) floating on the chloroform phase (iii) (see Figure S1a).

This droplet can then be deposited onto various substrates such as gold, glass, silicon, or PDMS (Figures 1b and S1b,c), for direct integration into microfluidics. As the residual supernatant evaporates, a ∼5 mm diameter densely packed random arrangement of AuNPs is formed (Figure 1c, SEM, and Figure S1e).

These MLagg films show distinct regions with a single layer (1ML) or double layer (2ML) of AuNPs (Figure 1d, bottom). The second layer forms because the surface area of the drying droplet (consisting of a single AuNP layer) is larger than its footprint on the substrate (due to surface pinning). We note that the relative areas of mono/bi-layer MLagg can be controlled by predefined surface patterning of the substrate. The 1ML and 2ML layer regions are clearly visible in brightfield (BF) and darkfield (DF) images as well as SERS map scans and atomic force microscopy profiles (Figure 1d, top and Figure S2). The plasmonically active nanogaps produce strong SERS signals from trapped molecules (Figure 1e) exhibiting stronger emission in the 2ML region. This enhanced optical interaction is confirmed by DF spectra showing distinct resonant modes from the 1ML and 2ML regions (Figure 1e). In both cases, the precisely controlled gap spacings (Figure 1e) produce clear plasmonic modes from the 1ML and 2ML AuNP films, which redshift and strengthen with increasing number of layers.

### Defining, Stripping, and Redefining Nanogaps

**Figure 2:**
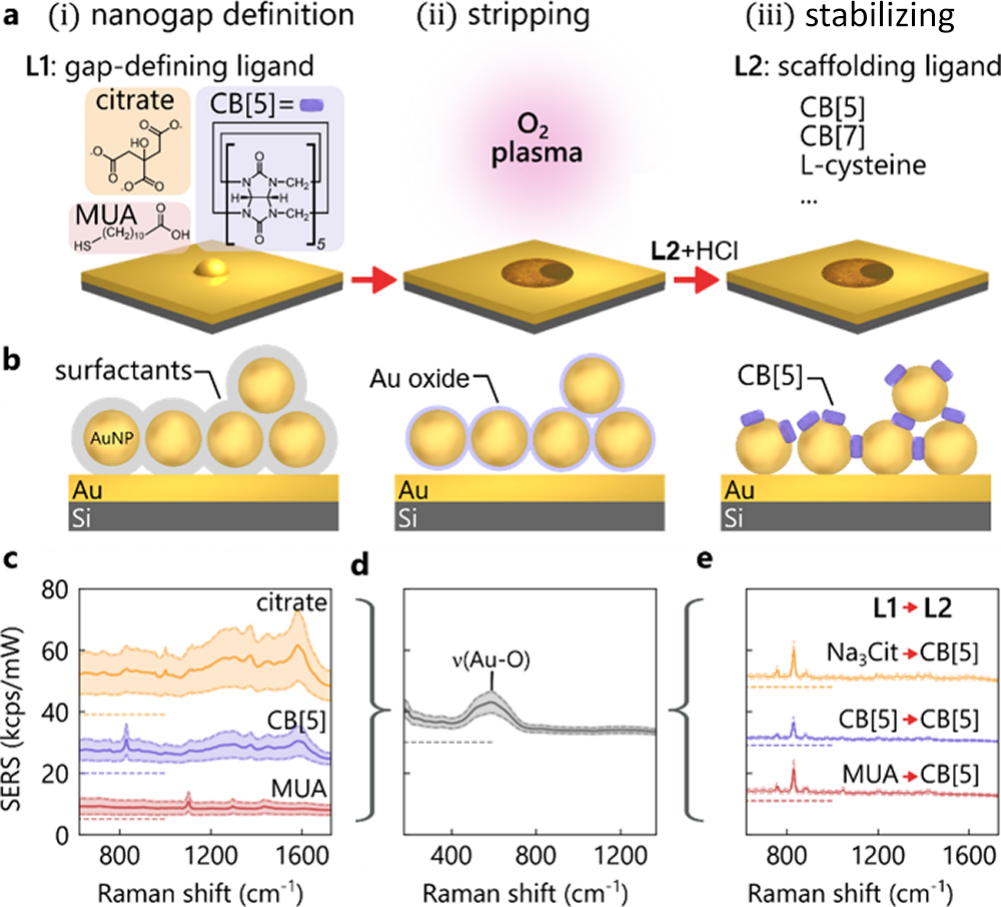
Nanogap definition, stripping, and redefinition. (a) Three-step nanogap redefinition protocol in MLagg films. (i) Initial surfactants (L1) define nanogaps, (ii) oxygen plasma strips out surfactants, and (iii) nanogaps stabilized using a second ligand (L2). (b) Corresponding surface modifications. (c–e) Stacked SERS spectra (not normalized) from spatial mapping of MLagg films recorded after each step (i–iii). Shading shows interquartile variation; dotted lines show the baseline shift.

A key feature of these close-packed MLagg films is the control of their very tight interparticle gap spacing. Since the films are formed on a substrate as a 2D layer with all their gaps accessible, it is now possible to introduce a process to transform the molecular scaffolding in each gap. This contrasts with aggregates in solution in which such molecular replacement is not viable. This three-step process (Figure 2a,b) separates (i) the definition of nanogap size by initial scaffolding, (ii) the stripping of all scaffold molecules, and (iii) the stabilization of the gaps using various species (with different chemistries). This makes it possible to fully control and fine-tune the nanogap spacing and facet chemistry.

The initial gap spacing is defined by the dimensions of the aggregating agents that act as gap-defining ligands. Using different aggregating agents, a range of interparticle spacings can be produced (0.9–3 nm). If the ligand of choice is not water-soluble, it can instead be dissolved in the organic chloroform phase which, after vigorous shaking of the two-phase system, binds to the AuNP surface. To demonstrate the nanogap definition, we compare the use of 11-mercapto-undecanoic acid (MUA), sodium chloride (NaCl), and cucurbit[5]uril (CB[5]) as the initial aggregating agents (Figure 2b, top).

**Figure 3:**
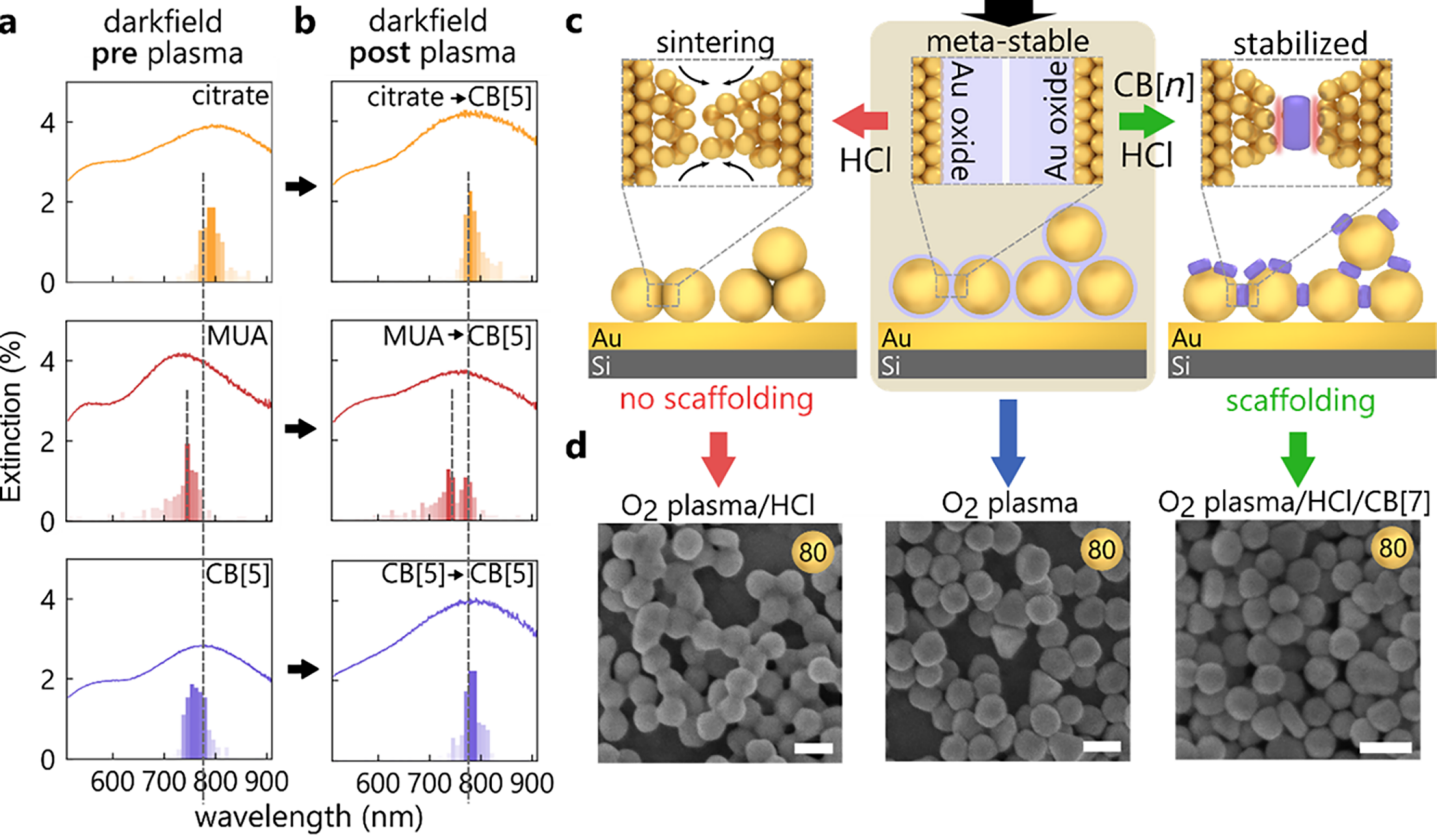
Controlled gold atom movement. (a) DF spectra of three ligands (citrate, MUA and CB[5]) before plasma treatment. Average spectrum from 100 positions, together with histograms of peak wavelength. (b) DF spectra of plasma-treated MLagg films after CB[5] re-scaffolding. (c) Illustration of gold atom movement after O_2_ plasma, leads to sintered or stabilized nanogaps in the absence/presence of a ligand. (d) SEM images of nanoparticle sintering (left) after plasma stripping (center) and when followed by direct acid treatment with stabilization molecules (right), scale bar 100 nm.

The SERS spectra recorded after the deposition and drying of the films (Figure 2c) reveal the nanogap chemistry of the initially prepared MLagg films. The NaCl-salted films (orange) show the citrate surface chemistry of the AuNPs employed (BBI). The characteristic vibration at 995 cm^–1^ shows that citrate anions define the gap spacing,^32^ which is estimated to be 1.0 ± 0.2 nm (shaded region shows the interquartile range over an area 200 μm × 200 μm, laser spot size ∼1 μm). Aggregating the MLagg films instead with CB[5] (blue) gives similar SERS spectra to the NaCl-salted films but with additional strong CB[5] modes, particularly the ring-breathing mode at 829 cm^–1^. This shows that the CB[5] does not fully displace citrate anions from the AuNP surfaces leading to a mixed chemical environment. The gap spacing here is constrained to the CB[5] portal-to-portal height of 0.9 nm (Figure 2c, purple). The SERS spectra of the MUA-aggregated films (red) show an even higher relative variance than the NaCl or CB[5] MLagg films. The long and flexible alkane chains (as compared to smaller citrate or rigid CB[5]) are expected to lead to larger gap sizes (compare DF spectra in Figure 3) and gap size variation.

The subsequent stripping step utilizes oxygen plasma cleaning of the MLagg films (450 W, 30 sccm, 30 min) to fully remove all molecules from the AuNP gaps and surfaces. The SERS spectra (Figure 2d) confirm this complete stripping of the surfactants, with all molecular vibrations now absent (see Figure S3a,b for X-ray photoelectron spectroscopy (XPS) spectra). Surprisingly, even after removal of the stabilizing ligands, the plasma-treated AuNP gaps remain stable and no sintering is observed (Figure 3d center). Oxygen plasma cleaning not only decomposes surface organics, but the process also introduces several monolayers of Au oxide on the AuNP surfaces, as evidenced by XPS measurements (Figure S3c,d) and seen directly in the SERS spectra which exhibit the well-known broad peak^33^ of ν(Au–O) ≃ 600 cm^–1^ (Figure 2d). The growth of the Au oxide layer in the nanogap saturates within 15 min of oxygen plasma (Figure S5a). The volume per Au atom doubles when forming the Au_2_O_3_ phase, implying that it expands into the gap until three surface layers are fully oxidized, filling the previously defined interparticle gap (as supported by analysis of the DF spectra, Figures S4 and S5). In the absence of stabilizing ligands, this Au oxide “plug” maintains the AuNP nanogaps, but it is important to note that Au oxide is known to be thermodynamically metastable and can spontaneously decompose back to Au(0), resulting in sintering. However, in air if kept at low temperature (<4 °C) and shielded from room light, the NP films can be maintained with the oxide layer for >24 h.^34,35^ In aqueous solution at neutral pH, the films maintain stability for several days if the salt concentration is minimized, in particular by ensuring Cl^–^ concentrations are low enough to prevent dissolution of the oxide layer through soluble Au(III) species.^36^

In the final step, a scaffolding ligand is reintroduced to plasma-cleaned AuNP surfaces by immersing the MLagg films in an appropriate ligand solution. A wide range of small ligands have been successfully tested, including L-cysteine, cysteamine, CB[5], CB[7], and others discussed below. We first show that irrespective of the initial ligand L1, it can be replaced with L2 = CB[5] which has highly advantageous scaffolding properties. Three initial L1 surfactants (MUA, CB[5], NaCl) are employed as above, before immersing the films in aqueous CB[5] solution (∼1 mM). At pH 7, several days are required for the CB[5] to penetrate all nanogaps because the Au oxide layer still fills the nanogap. However, at acidic conditions (pH < 3 using HCl or H_2_SO_4_), the Au oxide layer rapidly decomposes,^37^ allowing CB[5] to bind within seconds to the AuNP surfaces. All three films now show pristine CB[5] SERS spectra (Figure 2e), exhibiting only a small relative variance (hundred-fold smaller than before replacement). This confirms that the gap nanoarchitecture is indeed now much more consistent after the oxidation/replacement (SERS variance improved by >50-fold), implying its reconstruction into a reliable geometry as well as the removal of unwanted molecules. This universal reconstruction of nm-scale gaps is surprising.

To report the statistical significance of these results, the relative standard deviation (RSD) is calculated. This key factor characterizes the uniformity and reproducibility of the substrates and is defined as the standard deviation of peak intensities over their mean intensity.^38^ Two different RSD values are calculated, the global RSD which averages all of the spectra from across the entire MLagg film, as well as the local area RSD across a selected 600 × 600 μm area (3 × 3 grid of points). The initial SERS intensities give CB5, MUA, and citrate global RSDs of 45, 57, and 51% respectively, with values of 24, 26, and 25% after the scaffolding ligand is reintroduced. Regardless of the initial nanogap definition molecule, the global uniformity of the substrate improves in uniformity after replacement. The local RSD further highlights this: from initially 11, 13, and 22% (CB5, MUA, and citrate) it reduces after stabilization with CB5 to 8, 9, and 8%. From this, it can be concluded that high uniformity is achieved within areas of similar nanogap density, as well as highlighting the robustness of this cleaning and rescaffolding protocol.

### Controlled Restructuring of Nanogaps

Due to the difficulty of quantitative and reliable TEM characterization of sub-nm-scale gaps, a more suitable tool for analyzing these changes is DF spectroscopy. Spectra are collected over the same area across the three MLagg films, both prior to oxygen plasma treatment and just after the redefinition of the gap with CB[5] (Figure 3a,b). For each MLagg sample, histograms record the peak wavelength of the coupled plasmon mode, with the average DF spectra also shown. These peak wavelengths are determined by the gap sizes and the effective refractive index inside each nanogap.^39^

The DF spectra before plasma treatment for different initial ligands L1 are distinctively different in peak positions and distributions. The MUA film (red) exhibits the shortest wavelength plasmon (∼740 nm) and widest peak distribution. As MUA molecules are longer and more flexible (in comparison to citrate and CB[5]), this confirms a larger average gap size which fluctuates more (estimated as 1.3±0.4 nm^40^). The CB[5] and citrate MLagg films by contrast show a narrower peak distribution. The CB[5] peak is blue shifted (∼780 nm) in comparison to the citrate-defined nanogaps (∼800 nm), and assuming similar refractive indices, this difference suggests a smaller mean gap size for the citrate-defined MLagg film (by ∼0.1 nm).

Remarkably, despite these different initial gap sizes, after plasma treatment and re-scaffolding of the nanogaps with CB[5]/HCl, a peak wavelength at ∼790 nm is now seen in the DF histograms for all three MLagg films (Figure 3b). This indicates that the CB[5]-defined rescaffolded gaps have a consistent size and refractive index and also exhibit consistent SERS spectra (Figure 2e) after rescaffolding (see also Figures S4 and S5). The MUA film shows a slightly larger second peak at ∼735 nm after plasma treatment and re-scaffolding, suggesting that some larger gaps are also present although the SERS spectra (Figure 2e) show no sign of residual MUA molecules.

Additional experiments confirm the stabilization of nanogaps by various ligands/compounds after plasma treatment. Molecules such as 3-mercaptopropionic acid (MPA), citrate, acetic acid, cysteamine, 4-aminothiophenol (ATP), 4-mercaptobenzoic acid (MBA), 4-mercaptopyridine (MPy), cyclodextrin, dopamine, paracetamol, ethanol, and methanol all give robust constructs (Figure S7). By contrast, molecules that do not typically bind to gold such as acetone and glucose do not act as stabilizing agents.

### Surface Gold Atoms “Flowing” at Room Temperature

The DF and SERS data evidence atomistic restructuring of the gold surface inside the plasma-treated nanogaps, which makes it possible to redefine the nanogaps in a controlled way. After stripping organics from the AuNP surface during oxygen plasma treatment, an oxide layer is formed, stabilizing the metastable state by plugging the AuNP gaps (Figure 3, center). Even when immersed in CB[5] solution (pH 7), the oxide layer protects against CB[5] binding inside the nanogaps with only very weak (few %) CB[5] SERS peaks emerging over several days.

Upon addition of small quantities of 0.5 M HCl (pH 0.3), the Au oxide layer decomposes, which immediately destabilizes the AuNP architecture. We observe two pathways for this process: (1) if the MLagg films are exposed to HCl in the absence of any stabilizing ligand, individual gold atoms inside the nanogaps flow toward adjacent AuNP facets (Figure 3c, left) forming bridges between AuNPs and losing all SERS (within seconds). While AuNP sintering typically requires heating of the substrate to overcome the activation barrier for gold atom movement,^34,41−43^ the chemical sintering process here occurs at room temperature. We find that this process is irreversible; subsequent plasma treatment does not reactivate the MLaggs. (2) Conversely, in the presence of a ligand and HCl (Figure 3c, right), the binding of the ligand into the nanogaps effectively prevents the AuNPs from sintering. We thus suggest that a key component for this step is the simultaneous decomposition of the oxide layer and re-scaffolding of AuNPs with the ligand, which precisely reconstructs the nanogap. The mechanism for decomposition of the oxide layer upon treatment with HCl with and without ligands is under detailed investigation, but we note that Au dissolution can proceed through formation of soluble Au(III) complexes.^36^ The extent of Au atom loss during rescaffolding does not appear to adversely affect the optical properties, as we observe that plasma cleaning and rescaffolding cycles can be repeated at least seven times on the same AuNP film while preserving SERS activity (Figure S6).

The results of the HCl-induced sintering for freshly plasma treated AuNPs can be observed in SEM images (Figure 3d, left). These images clearly show that the gold atoms on facets of adjacent AuNPs flow toward each other, forming bridges. For larger AuNPs (diameter 100 nm) which have larger initial facets,^44^ this sintering is less pronounced than for smaller AuNPs (60, 80 nm) (Figure S8). Most important to note is that sintering neither occurs for non-plasma treated MLaggs exposed to the same concentration of HCl (Figure S9a) nor for the plasma and ligand/HCl-treated films (Figure 3d, right). Repeating the sintering experiment with the same molar concentration of H_2_SO_4_ instead of HCl leads to a similar outcome (Figure S9b). The size dependence observed suggests that curvature can drive this process, in concert with liquid–solid surface energies.

### MLagg Films as Molecular Sensors

Plasma-treated MLagg films open up wide opportunities for molecular sensing applications. We now show how MLaggs offer improved spatial reproducibility after plasma cleaning in conjunction with full control over the nanogap ligands, allowing for stripping off unwanted compounds from the metal surfaces (such as citrate). With CB[*n*] as the scaffolding molecule, we find that substrates can be multiply reused by immersing them in 1 M HCl solution.

### Aqueous Solution Sensing

To demonstrate these sensing and cleaning capabilities, we first show that toluene, a very hydrophobic and volatile compound, can be detected down to concentrations below 10 ppm. In order to simplify handling and better control the toluene concentration, we first detect a range of different concentrations in an aqueous solution. Despite its hydrophobicity, it is possible to obtain concentrations in water up to 5 mM which is sufficient to cover the desired range.

**Figure 4:**
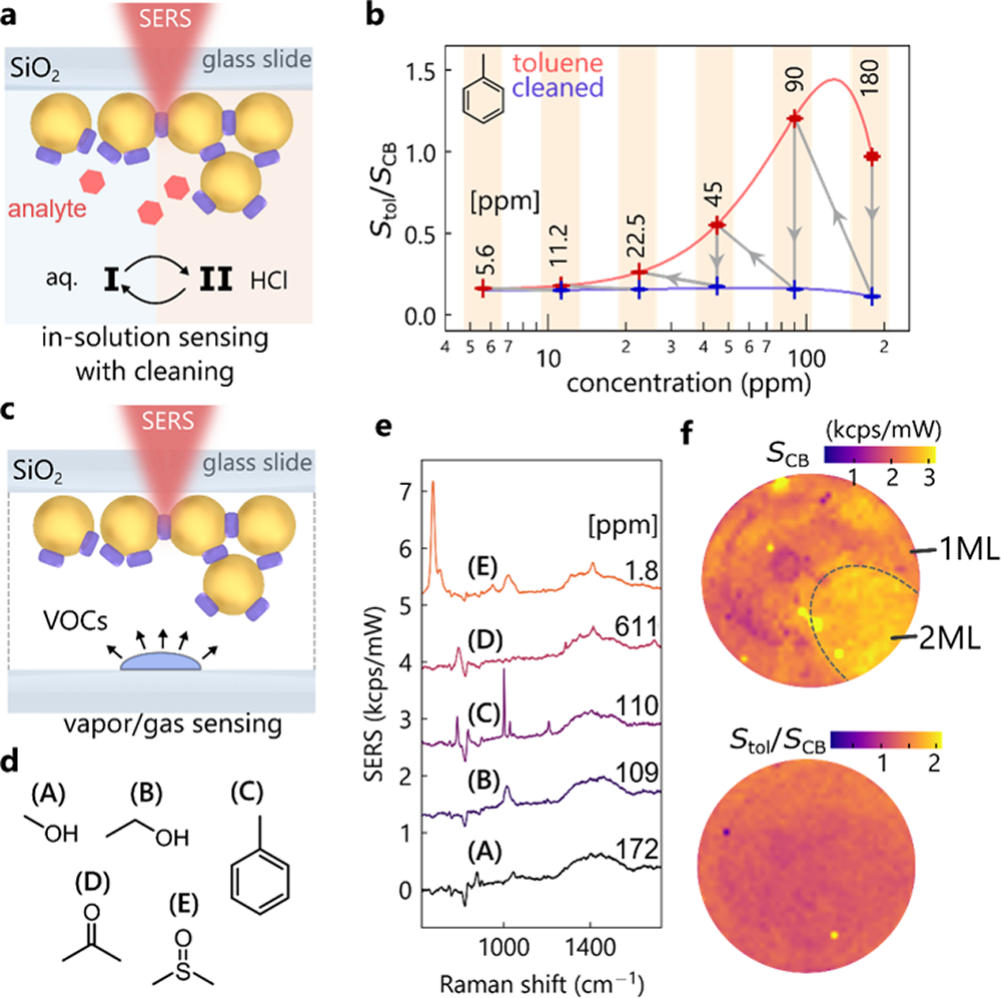
Sensing capabilities of treated MLagg films. (a) Sensing setup for recyclable sensing of hydrophobic toluene. (b) Extracted toluene signature peak (995 cm^–1^) normalized to CB[7] scaffold, showing low detection limits. (c) Sensing protocol for volatile organic compounds (VOCs) in a sealed container. (d) VOCs (methanol, ethanol, toluene, acetone, and dimethyl sulfoxide) used for experiments. (e) SERS spectra of VOCs (with CB[7] signal subtracted). (f) SERS maps showing CB[7] signal peak intensity (top) and toluene signature peak normalized to CB[7] (bottom).

The experimental protocol (Figure 4a) cycles between (I) exposing a MLagg sample to toluene (20 min) and (II) subsequent cleaning with HCl followed by blow-drying with N_2_. The MLagg film used for this experiment is deposited on a thin glass cover slip, plasma-cleaned, and re-scaffolded with CB[7] molecules. The SERS signals are collected through the cover slip, which is a key advantage of these multilayer films that combines simple optics with immersion in liquid or vapor cells.

Extracting the ratio of the toluene ring-breathing mode (995 cm^–1^) (see Figure S10) to the CB[7] signature peak (833 cm^–1^) clearly demonstrates the cleanability with HCl as well as a detection limit below 10 ppm (Figure 4b). This is below the ACGIH 8-hour toluene exposure threshold limit of 20 ppm. It is important to stress that the same MLagg was used throughout this experiment starting with the highest (180 ppm) concentration of toluene. The small background signal ratio after each cleaning is nearly constant, increasing only after the first exposure, possibly due to slight restructuring of the gold. At the highest concentrations, the SERS signal is reduced, a common trend observed in SERS, attributed to the dipole depolarization effect occurring at dense analyte coverages (Figure S11).^45,46^ For the nonplasma cleaned films, this effect is not observed, and the detection limit is worse (Figure S11).

### Vapor Sensing

To demonstrate the sensitivity of MLaggs to a range of volatile compounds, films are exposed to the vapors of five molecules (Figure 4c,d), again on plasma-cleaned and CB[7] re-scaffolded MLaggs. Sensing is performed in a glass container sealed by the coverslip, which allows the vapor to build up and reach its saturation concentration (see Table S1 for vapor concentrations). The background-subtracted SERS intensities (Figure 4e) confirm the capability of MLagg films to detect methanol, ethanol, toluene, acetone, and dimethyl sulfoxide (DMSO). Here, CB[7] is employed as it has a large enough inner volume to trap each of these molecules.^29^ DMSO produces the strongest SERS signals at a very low saturation concentration of just 1.8 ppm, which is likely because DMSO interacts most strongly with the gold surface.^47^

The spatial distribution and repeatability of toluene vapor sensing inside the CB[7]-defined nanogaps on the multilayer aggregates are tracked through high resolution 50 × 50 μm SERS maps (Figure 4f). The CB[7] vibrational response clearly images the monolayer (weaker), bilayer (stronger), and mixed ML regions on the films. Essentially, this maps the number of gaps under the laser spot, which thus can be used as a normalization signal. Comparing the ratio of toluene signal normalized to CB[7] (Figure 4f, bottom) reveals a much more homogeneous response, independent of the number of nanoparticle layers or gap density. This suggests that toluene vapor penetrates equally deeply into the nanogaps of both layers. Quantitative measurements can thus reliably use the normalization to CB[7] vibrational modes to calibrate analyte signals. Ascertaining the ultimate limits of detection for VOCs with MLagg films requires systematic experiments to examine the optimum re-scaffolding ligand L2 for each analyte. However, the repeatable and sensitive performance of VOC sensing suggests their future utility.

### Flow Sensing and Cleaning

**Figure 5:**
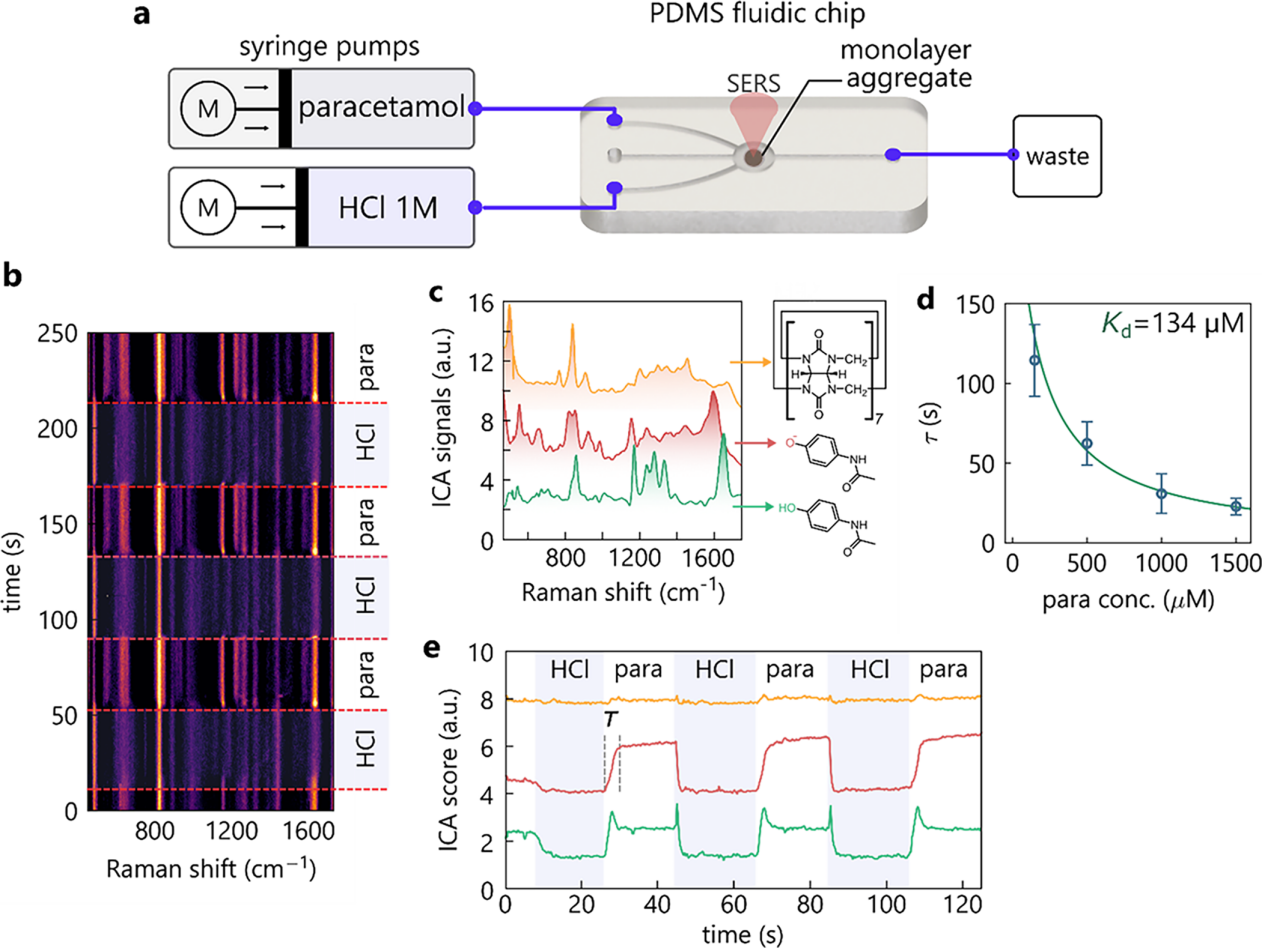
Flow Sensing. (a) Cycling SERS sensing of paracetamol in a PDMS flow-cell. (b) Time-resolved SERS measurements showing paracetamol (para) and acid-cleaned (HCl) spectra. (c) Extracted independent components resemble CB[7], protonated and deprotonated paracetamol. (d) Equilibration times for different paracetamol concentrations with fit (line) and standard error. (e) Time evolution of protonated and deprotonated paracetamol components during cycling (colors as in c).

A further application of MLaggs is their direct integration into flow cells for in-flow sensing of analytes (Figure 5a). As for the liquid and vapor sensing, MLagg films are tethered to a glass coverslip (coated with a 5 nm Cr layer to increase adhesion, see Methods) and plasma-bonded to PDMS fluidic chips. Again, the MLaggs are plasma cleaned and re-scaffolded with CB[7]. Analyte flow ∼10 μL/s is initiated and controlled by two syringe pumps which are connected to the PDMS chip. The SERS pump laser is incident through the cover slip with light collected along the same path, straightforwardly separating optics and fluidics.

With this flow-cell, we investigate the kinetics of analyte sequestration and cleaning of the nanogaps. This is demonstrated in flow by switching the liquid flowing over the multilayer films between a selected analyte and HCl for cleaning. In this instance, paracetamol is chosen as the analyte. During this experiment, the cleaning cycle with HCl for 20 s is followed by flowing paracetamol (1.5 mM) for another 20 s. The resulting kinetic SERS scan using 0.5 s integration times clearly resolves the switching between the flowing paracetamol and HCl (Figure 5b). We find tens of cycles of cleaning and sensing retain consistent signals, with the paracetamol SERS achieving a RSD of 5.7% between cleaning cycles.

Extracting the different component spectra from such dynamic measurements is ideally suited to independent component analysis (ICA), which here retrieve three independent spectra (Figure 5c) (see Methods). These spectra resemble CB[7] and paracetamol. The latter shows two different spectra which are related to its protonated and deprotonated states (from DFT). The corresponding time-dependent ICA scores (Figure 5e) reveal that cleaning with HCl occurs within a few seconds and is fully repeatable between cycles. Furthermore, it is evident that the time-dependent sequestration of paracetamol into the sensing gaps follows an exponential function (Figure 5d), which matches a simple theoretical model based on Langmuir isotherms. This confirms that equilibrium can be obtained in this simple and open multilayer nanoparticle geometry despite the small gap sizes used to obtain intense SERS for sensing.

The protonated paracetamol profile exhibits sharp spikes just after the paracetamol flow commences as well as when the HCl flow is initiated. During the transition from HCl to paracetamol, a significant fraction of protonated paracetamol enters the nanogaps. The protonation occurs because of acid back-flow into the paracetamol-carrying tubing during the HCl flow. As more paracetamol flows over the multilayer film, the pH recovers to equilibrium, resulting in a fixed protonated to deprotonated signal ratio. During the transition from paracetamol to HCl, the acid flow first protonates the paracetamol inside the nanogaps before it is released, leading to the second observed spike. This clearly demonstrates that protonation is faster than the removal of paracetamol from the nanogaps, as expected from the diffusion rate dependence based on the proton to paracetamol molecular weights. This rapid SERS flow sensing device is therefore very promising for distinguishing a wide range of small-molecule analytes.

## Conclusions

MLaggs composed of random close-packed films of one (or several) layers of gold nanoparticles offer a sensing platform with excellent optical and fluidic access. We show here that their nanogap chemistry can be controlled far more carefully than previously, which is vital for real sensing applications. Treating the films with an oxygen plasma strips all organic compounds off the surface whilst leaving the gold facets intact. The oxide layer remaining on the surface protects and stabilizes the nanoparticles from sintering. If removed by acid without any ligands present, gold atoms flow between opposite facets forming bridges that destroy the sensing properties. However in the presence of a ligand, the gold facets restructure to accommodate these new scaffolds inside the nanogaps, modifying the local chemical environment. Even with initially nonuniform nanogaps defined by various molecules, it is possible to successfully incorporate CB[5] or other molecules into the nanogaps after oxygen plasma and acid treatment. The newly re-scaffolded films now deliver highly reproducible SERS spectra with robust and precise gaps (as for solution aggregation, but now attached to a solid support).

This facile protocol gives a reconfigurable and sensitive SERS substrate with excellent sensing capability for compounds in solution (such as toluene) and vapors. Cleaning of the MLaggs between sensing cycles is achieved by flowing HCl over them. Plasma-treated CB[7] MLaggs detect many volatile organic compounds including DMSO, toluene, acetone, methanol, and ethanol. VOCs penetrate both monolayers giving a calibrated analyte response using normalization by CB[7] signals. The MLaggs are shown to be exceptionally suitable for integration into flow cells due to backside optical access, and demonstrate repeatable sensing and cleaning cycles. This work thus offers the prospect for continuous monitoring in many applications, such as water quality, urine, or saliva sensing, spanning from environmental to healthcare monitoring.

## Methods

### Multilayer Aggregate Preparation

Equal volumes (500 μL) of chloroform (CHCl_3_) and commercial AuNPs (BBI Solutions) are added to a standard 2 mL centrifuge tube (Eppendorf) forming a two-phase system with the AuNP suspension floating on top of the chloroform phase. The AuNP size is 80 nm for SERS measurements at 785 nm excitation if not otherwise noted. For the aggregation step using CB[*n*], 5 μL of a ∼1 mM CB[*n*] solution is mixed with the AuNP phase. For NaCl aggregation, a higher volume of 150 μL of a 0.5 mM NaCl solution is added. Aggregation using 11-mercaptoundecanoic acid (MUA) is achieved by saturating the chloroform phase with MUA followed by vigorous shaking of the centrifuge tube. By this method it is possible to aggregate AuNPs with molecules that are not soluble in water but in chloroform (such as MUA). Each aggregating agent has been optimized to account for the different charge stability it imposes and their impact on the aggregation of the AuNPs.

After vigorous shaking of the AuNP/chloroform system, the goal is to remove ∼80% of the supernatant of the freshly aggregated AuNP via careful pipetting. This step is followed by replenishing the centrifuge tube with DI water. Replacing of the supernatant with DI water is repeated three times to remove large aggregates and to reduce salt concentrations significantly. During this process, a monolayer of AuNPs is formed between the liquid-air and liquid-chloroform interfaces (extending up the inside walls of the centrifuge tube). Finally, as much as possible amount of supernatant is removed (>80%) to concentrate the AuNP monolayers into a small droplet (1–5 μL). This droplet is then transferred onto a substrate such as a gold-coated Si-wafer or a coverslip (for flow and vapor sensing experiments). The droplet is left to dry for several hours.

Once dry, excess salt is removed by gentle rinsing with DI water followed by blow-drying with N_2_. Plasma treatment is performed with a commercial oxygen plasma cleaner (Diener electronic GmbH + Co. KG) for 30 min at an oxygen mass flow of 30 sccm at 90% RF power for 30 min. CB[*n*] re-scaffolding is achieved by firstly drop-casting a CB[*n*] solution (∼20 to 50 μL, 1 mM) onto the MLagg films, then, adding a small amount (1–5 μL) of HCl (1 M) to the CB[*n*] droplet. After 10 min, the MLagg films are rinsed with DI water and finally carefully blow-dried.

### Dark-Field and SERS Measurements

SERS spectra are taken on a commercial Raman instrument (Renishaw inVia) at 785 nm excitation (laser line profile) using a 20× objective at ∼150 μW of laser power (0.1% setting) to avoid damage to the MLagg films. MUA, CB[5], and NaCl high-resolution maps are recorded using supplied Renishaw software. For map scans, several MLaggs are deposited onto one large gold-coated coverslip (with 5 nm chromium adhesion layer) each resulting in 2–4 mm diameter films. SERS spectra are taken with a 200 μm grid size (10–20 rows and columns, depending on film diameter) exposing the sample for 1 s per spectrum.

DF scattering spectra are taken in reflection on a custom microscope setup consisting of an Olympus BX51, a 20× Zeiss objective and an Ocean Optics QE-Pro spectrometer (0.5 s integration time). All spectra are referenced to a white light scattering target (Labsphere). Grid size is consistent with SERS measurements (200 μm). To obtain alignment between SERS and dark-field spectra, the substrates are spatially referenced.

### SEM Measurements

MLagg samples are prepared according to the standard protocol (see Multilayer Aggregate Preparation) and deposited on Au-coated silicon wafers which are cut into small pieces. SEM measurements are taken on a FEI Philips Dualbeam Quanta 3D SEM (dwell 100 ns, HV 5 kV, current 25 pA and WD ∼4 mm). Magnification is varied between 150, 200 and 250 k.

### XPS Measurements

X-ray photoelectron spectroscopy (ThermoFisher Escalab 250Xi) is used to measure the surface chemistry of aggregated AuNP films using a monochromated Al Kα X-ray source. Survey spectra are recorded with a pass energy of 100 eV and high-resolution spectra of elemental peaks with 50 eV pass energy. The data were fit using the CasaXPS software (Casa Software Ltd., USA); spectral components were fit using Gaussian–Lorentzian functions.

### Vapor Sensing Experiments

A droplet of each volatile compound (∼50 μL) is pipetted inside a ∼5 mL glass vial whose rim and neck (inside and outside the vial) are wrapped with a single layer of “Parafilm M”. The CB[*n*] re-scaffolded MLagg deposited on a thin borosilicate coverslip (Menzel #1.5) is then placed on top of the glass vial with the MLagg facing the inside. To seal the setup, the coverslip is gently pressed against the “Parafilm” lined rim. To give sufficient time for the saturation concentration to build up, the setup is left for ∼20 min before SERS measurements are taken, through the coverslip. During the experiments, the droplets never fully evaporate indicating that there is sufficient analyte available to always build up the saturation concentration. Saturation concentrations are listed in Table S2.

### Aqueous Solution Sensing Experiments

Aqueous toluene sensing is performed in a similar fashion to vapor sensing. The CB[7] re-scaffolded MLagg is again deposited on a thin borosilicate coverslip (Menzel #1.5) followed by placing it on a droplet of a toluene solution for 20 min (±10 s) after which a SERS spectrum is taken immediately. For the experiment, only one film is used which is recycled between measurements by HCl treatment, rinsing with water as well as N_2_ blow-drying. The experiment starts with the highest toluene concentration and moves to lower concentrations after each cycle.

### Flow Sensing Experiments

The paracetamol/HCl sensing and cleaning experiments are carried out on a MLagg film deposited on a coverslip (Menzel #1.5). The MLagg films are prepared according to the standard protocol but re-scaffolded with CB[7] molecules instead of CB[5]. The films on the coverslip are then plasma bonded to a PDMS chip (AuNP films face inside the channels of the chip) by a short exposure (a few seconds) to oxygen plasma. Briefly, the PDMS chip is manufactured by standard soft lithography from a template master mold. For the process, the SU-8 2100 negative photoresist is spin-coated evenly onto a silicon wafer to yield a layer height of ∼150 μm. After additional baking and exposure to UV light (through photomask), the photoresist is developed by immersing the wafer into PGMEA (1-methoxy-2propanol acetate) and then hard baked. A PDMS kit (SYLGARD 184, Sigma-Aldrich) with a mixing ratio of 1:10 (curing agent to PDMS monomer base) is used to manufacture the chips (baking for 30 min at 120 °C after degassing). For flow experiments, two inlets and one outlet are punched (1 mm diameter) into the PDMS chip. The inlets are connected to custom-made syringe pumps containing HCl (1 M) and paracetamol (1.5 mM) solutions. Kinetic SERS measurements are taken through a 63× (NA = 1.2) water-immersion objective with 0.5 s integration time. The liquid flow is cycled between paracetamol and HCl for 20 s each.^48^

## Supporting Information Available

Images of AuNP aggregate formation, atomic force microscopy image, Xray photoelectron spectrum DF and SERS of MLagg films; repeated cleaning cycle of MLagg; SERS of MLagg with various example molecular scaffolds; SEM images of AuNP size dependence and sintering with different protocols; calculated saturation concentrations of five volatile compounds; sensing of Toluene, chemical structure, SERS, and characteristic vibrations; and characterization of sensing toluene (PDF)
