# Supplementary material for: Controlling Atomic-Scale Restructuring and Cleaning of Gold Nanogap Multilayers for Surface-Enhanced Raman Scattering Sensing
Source: ACS Sens. 2023 Jul 6;8(7):2879–88. doi: 10.1021/acssensors.3c00967 (PMC10391707; doi:10.1021/acssensors.3c00967)
Supplement: Supplementary file 1 — se3c00967_si_001.pdf [file se3c00967_si_001.pdf]

Supplementary Information

Controlling atomic-scale restructuring and cleaning of gold nanogap multilayers for SERS sensing

David-Benjamin Grys[1], Marika Niihori[1], Rakesh Arul[1], Sarah May Sibug-Torres[1], Elle W. Wyatt[1], Bart de Nijs[1], Jeremy J. Baumberg[1*]

[1] NanoPhotonics Centre, Cavendish Laboratory, Department of Physics, JJ Thompson Avenue, University of Cambridge, Cambridge, CB3 0HE, United Kingdom

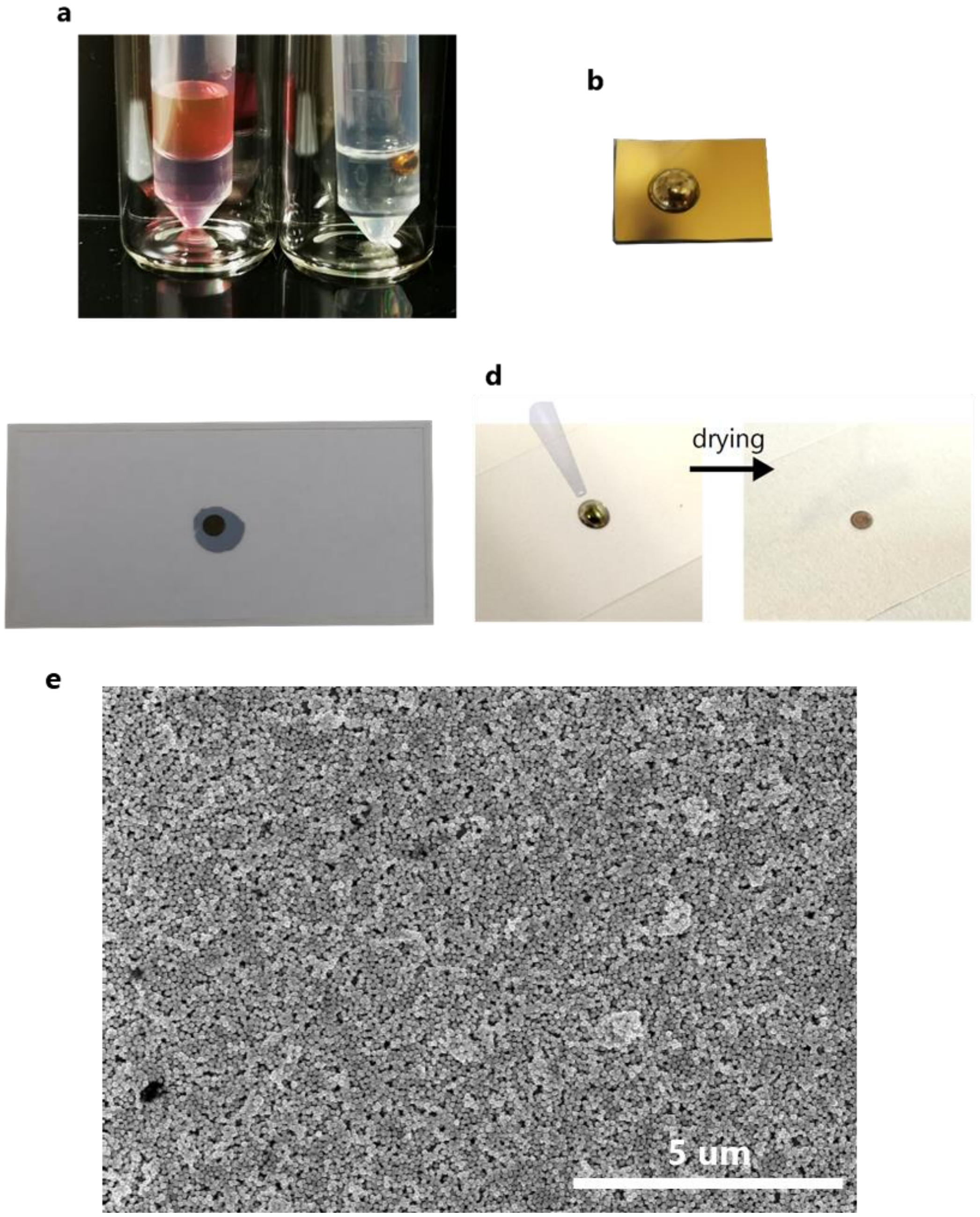

Figure S1. (**a**) AuNP colloid phase above chloroform (left), and concentrated AuNP droplet before deposition (right). (**b**) Deposited droplet on gold-coated substrate during drying. (**c**) MLagg (appearing black) deposited on coverslip (with chromium layer for improved adhesion appearing grey). (**d**) Direct deposition into coverslip. (**e**) Full size SEM image of Fig1.c from the manuscript of the MLagg.

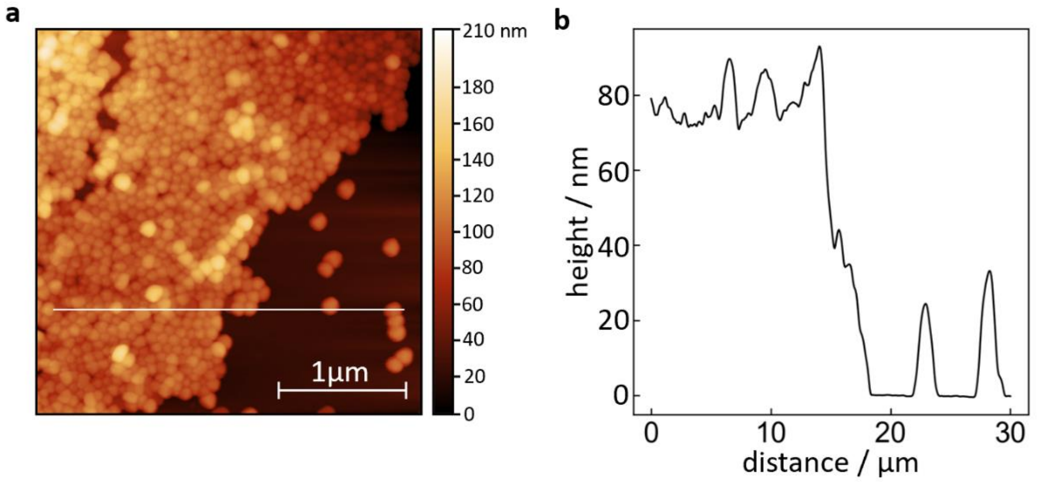

Figure S2. Atomic force microscopy (AFM) of 80 nm AuNP MLagg. (**a**) AFM image of the profile of the MLagg and (**b**) line scan displaying the stepping occuring at the interface between the substrate and the MLagg, along the white line in (a). Height corresponds with 1ML.

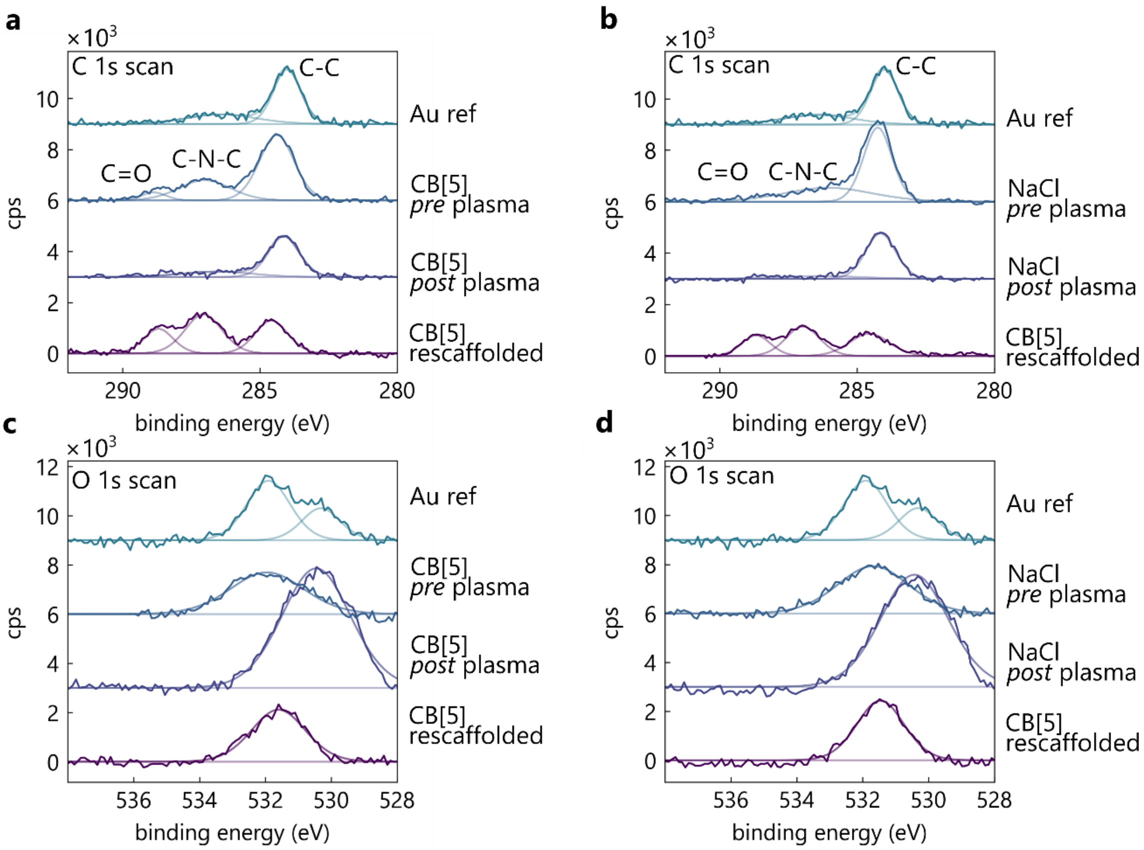

Figure S3. (**a**) Xray photoelectron spectrum (C 1s scan) of MLagg. Aggregates created using CB[5] show C=O and C-N-C bonds of CB[5] (*pre* plasma) which are removed after plasma treatment (*post* plasma). Characteristic bonds return after re-scaffolding with CB[5]. C-C contamination is present throughout measurements as well as bare gold surface (Au ref). (**b**) XPS spectrum (C 1s scan) of MLagg film created by NaCl aggregation (citrate stabilised) showing full removal of citrate and rescaffolding with CB[5]. (**c**) and (**d**) show evidence for oxygen on AuNP surface following plasma treatment.

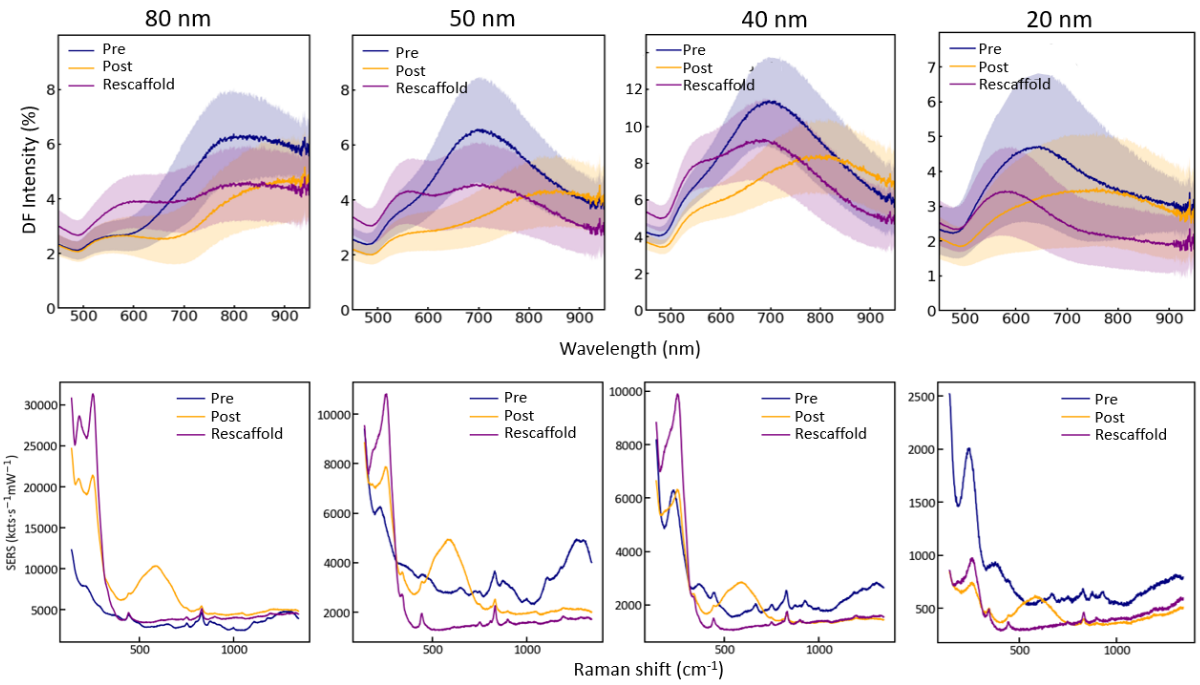

Figure S4. Dark-field scattering (top) and SERS (bottom) of MLagg films constructed from D=20,40,50,80 nm diameter AuNPs and aggregated initially by CB[7]. Pre = before plasma cleaning, Post = after plasma cleaning for 45 mins, Rescaffold = after HCl + CB[7] treatment.

The dark-field spectral peak positions of the MLagg fractal modes are closely related to the plasmons on a 1D chain. Their resonance wavelength can thus be estimated through an electrical coupling model (which resembles a tight-binding interaction model), giving $\omega^2 = \omega_0^2 - 2c^2 \cos\left(\frac{\pi}{N+1}\right)$ for N coupled NPs, isolated plasmon frequency ω_0, and coupling c. This allows the chain resonance to be directly related to the dimer mode resonance.

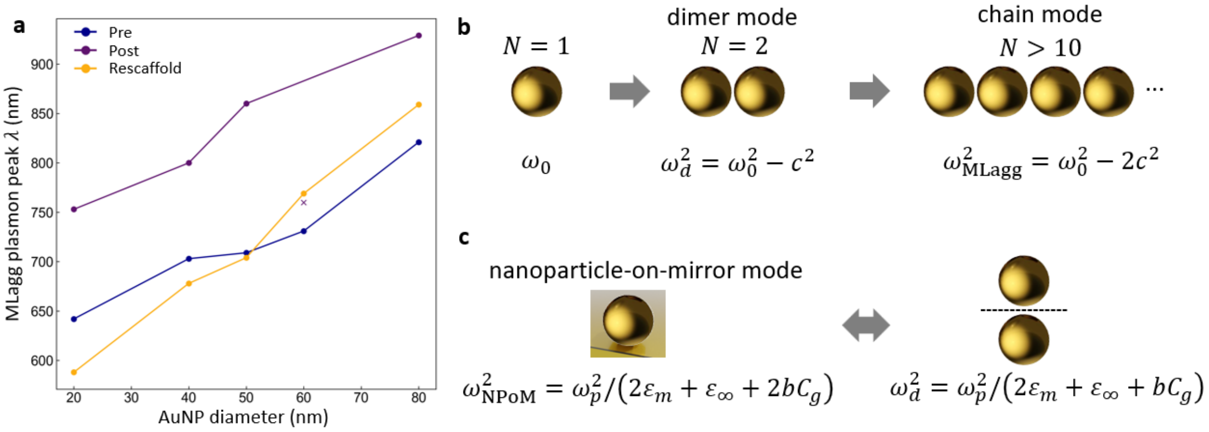

Figure S5. (**a**) Dark-field spectral resonance positions *vs* Au NP diameter (extracted from Fig.S7). (**b**) Spectral resonances of single NP, dimer, and chain of NPs in nearest-neighbour coupling approximation. (**c**) Relation between dimer and nanoparticle-on-mirror (NPoM) mode resonance.

A simple estimate to compare the dimer with the nanoparticle-on-mirror mode (which inserts a ground plane (i.e. mirror) halfway between the two NPs of the dimer) uses the factor of two scaling between their coupling capacitance.

Combining these gives the NPoM resonance in terms of the MLagg resonance as

$$\lambda_{\mathrm{NPoM}} = \sqrt{4\left(\lambda_{\mathrm{MLagg}}^{-2} + \lambda_0^{-2}\right)^{-1} - \lambda_p^2(2\varepsilon_m + \varepsilon_\infty)}$$

where λ_p~135 nm is the Au plasma frequency, ε_m=1 is the dielectric permittivity of the surrounding medium, ε_∞~8 is the short-wavelength Drude permittivity of Au, and λ_0~520 nm is the plasmon resonance of the Au NPs. Using the known dependence of the NPoM resonance on NP diameter, facet size (~20% of diameter), NP gap refractive index n_g, and gap size d allows the latter two values to be estimated (see [6] and https://www.np.phy.cam.ac.uk/npom-calculator). Using the data in Fig.S8(a), suggests that for CB-spanned gaps, d~0.9 nm and n_g~1.1 (as expected for a non-polar molecule). When they are oxidised, the significant red-shift is consistent with the expected refractive index of Au_2O_3 n_g~1.8 and compatible with the doubled gap size of d~1.8 nm. These values are consistent with ellipsometry measurements on planar Au surfaces, although the dielectric permittivity of Au oxide is not consistent in the literature.

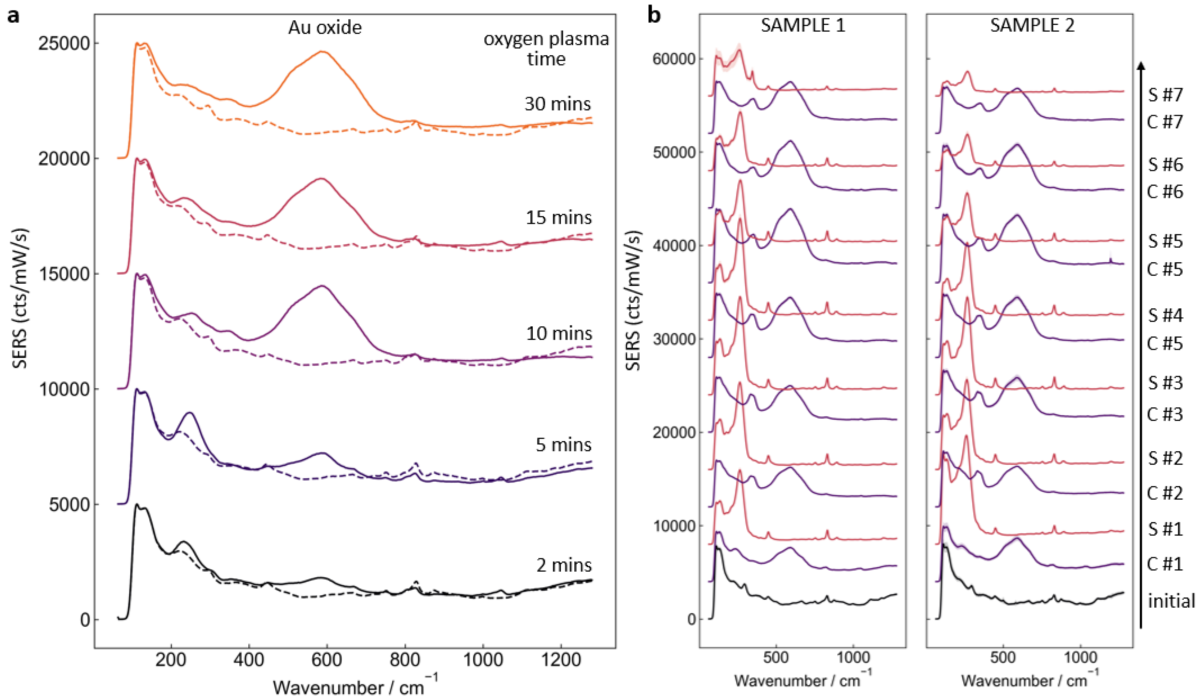

Figure S6. (**a**) SERS of 60 nm MLaggs formed by CB[7] aggregation, before/after 2-30 mins oxygen plasma cleaning showing the gradual oxidation of the nanogaps. (b) Repeated cleaning cycles of two MLagg samples, in each case formed from 60 nm AuNPs. Each cleaning cycle uses 30 mins of oxygen plasma cleaning, and re-scaffolding with 0.5 M HCl and CB[6].

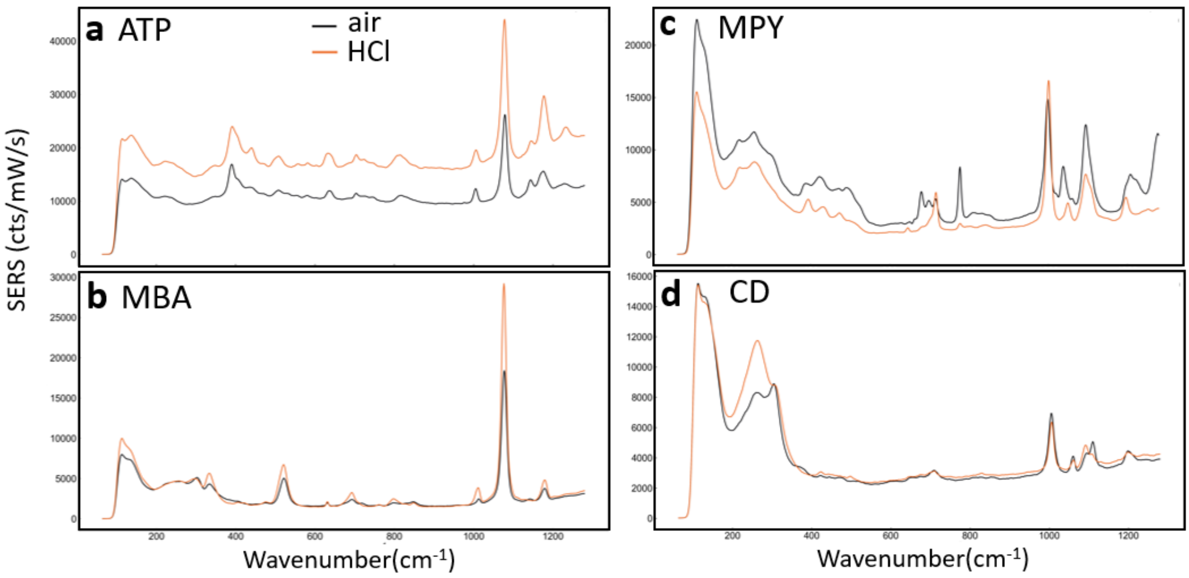

Figure S7. SERS of MLagg after oxygen plasma cleaning and re-scaffolding with various example molecular scaffolds. (**a**) 4-aminothiophenol (ATP), (b) 4-mercaptobenzoic acid (MBA), (c) 4-mercaptopyridine (MPy), and (d) cyclodextrin (CD). Spectra are taken after exposure to air, and after immersion for 10 mins in 1 M HCl.

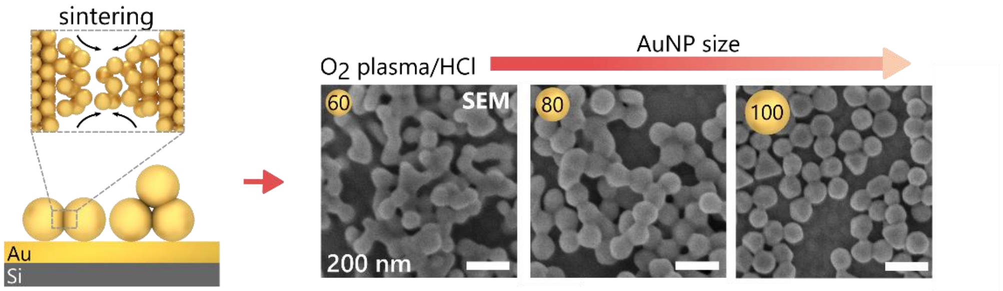

Figure S8. Gold nanoparticle size dependence of sintering (60, 80 and 100 nm commercial AuNPs). For the same concentration of HCl, larger AuNPs are more robust to sintering. SEMs are taken after plasma cleaning and treatment with HCl.

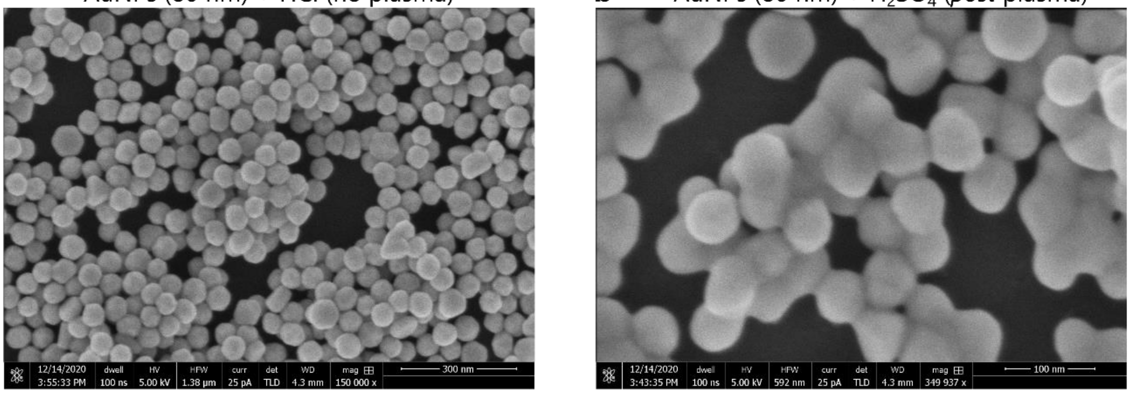

Figure S9. (**a**) Control measurement demonstrating that HCl exposure of non-plasma treated MLagg films does not lead to sintering. (**b**) Sintering of MLagg films after plasma treatment followed by exposure to H_2SO_4.This shows that not only HCl causes sintering.

sVOC	P/Pa ~ 20'C	c/mM	c/ppm
Acetone [1]	25544	10.52	610.8
MeOH [2]	2889.7	1.19	119.6
Toluene [3]	55.6	0.023	1.8
EtOH [4]	13010.9	5.36	171.6
DMSO [5]	5726	2.36	108.6

Table S1. Calculated saturation concentrations of five volatile compounds [1-5]

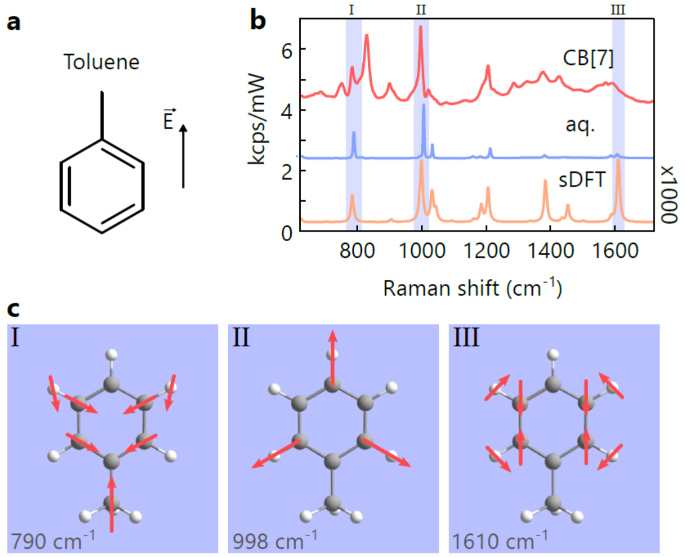

Figure 10. (**a**) Toluene chemical structure. (**b**) SERS of CB[7] re-scaffolded MLagg films and Raman of toluene solution (top), DFT calculation of toluene (centre) and polarised DFT (recalculated SERS intensities within polarised E-field, bottom) . (**c**) Normal modes of the characteristic vibrations.

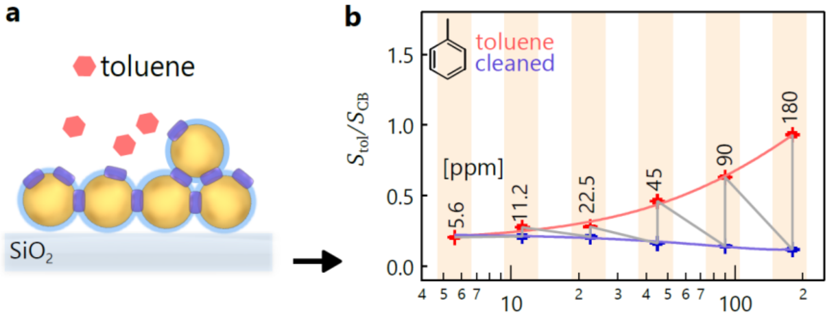

Figure S11. (**a**) Illustration of toluene experiment with MLaggs that were neither re-scaffolded nor plasma cleaned. (**b**) Toluene concentration series starting from highest concentration, followed by repeated HCl cleaning.

For the plasma-cleaned films in Figure 4 of the main text, the highest concentration (180 ppm) shows the toluene peak in a different spectral position (and in the same position as pure toluene). At lower concentrations, the peak is slightly shifted to lower wavenumber, as known for toluene interactions with water, indicative of a transition from dense to sparse coverage.

References

[1] D. Ambrose, C. H. Sprake, and R. Townsend, “Thermodynamic properties of organic oxygen compounds XXXIII. The vapour pressure of acetone,” *J. Chem. Thermodyn.*, vol. 6, pp. 693–700, 1974.
[2] L. M. Besley and G. A. Bottomley, “Vapour pressure of toluence from 273.15 to 298.15 K,” *J. Chem. Thermodyn.*, vol. 6, pp. 577–580, 1974.
[3] T. B. Douglas, “Vapor Pressure of Methyl Sulfoxide from 20 to 50°. Calculation of the Heat of Vaporization,” *J. Am. Chem. Soc.*, vol. 70, pp. 2001–2002, 1948.
[4] H. F. Gibbard and J. L. Creek, “Vapor Pressure of Methanol from288.15 to 337.65K,” *J. Chem. Eng. Data*, vol. 19, pp. 308–310, 1974.
[5] D. Ambrose and C. H. Sprake, “Thermodynamic properties of organic oxygen compounds XXV. Vapour pressures and normal boiling temperatures of aliphaticalcohols,” *J. Chem. Thermodyn.*, vol. 2, pp. 631–645, 1970.
[6] Elliott, E. *et al.* “Fingerprinting the Hidden Facets of Plasmonic Nanocavities”. *ACS Photonics* 9, 2643 (2022)
